# The use of complementary and alternative medicine during pregnancy: a cross-sectional study from Palestine

**DOI:** 10.1186/s12906-021-03280-8

**Published:** 2021-04-01

**Authors:** Yara Quzmar, Zeina Istiatieh, Hala Nabulsi, Sa’ed H. Zyoud, Samah W. Al-Jabi

**Affiliations:** 1 Department of Medicine, College of Medicine and Health Sciences, An-Najah National University, Nablus, 44839 Palestine; 2 Poison Control and Drug Information Center (PCDIC), College of Medicine and Health Sciences, An-Najah National University, Nablus, 44839 Palestine; 3 Department of Clinical and Community Pharmacy, College of Medicine and Health Sciences, An-Najah National University, Nablus, 44839 Palestine; 4 Clinical Research Centre, An-Najah National University Hospital, Nablus, 44839 Palestine

**Keywords:** Complementary and alternative medicine, Pregnant, Women, Palestine

## Abstract

**Background:**

The use of complementary and alternative medicine (CAM) is increasing worldwide. To the best of knowledge, there is a lack of studies that assess CAM use by Palestinian women during pregnancy. This research aims to determine the prevalence of Palestinian women’s use of CAM during pregnancy, the most frequently used CAM products during the pregnancy period, the main sources that encourage the use of CAM among pregnant women, and the causes of CAM use.

**Methods:**

A descriptive, cross-sectional study was conducted in Palestine using a questionnaire from April 2018 to March 2019. The samples were selected by convenience sampling, including currently pregnant or previously pregnant women in the Obstetrics and Gynecology (OBGYN) clinic at Rafedia Hospital and in primary health care clinics in Nablus city-Palestine. The questionnaire covered socio-demographic variables, health status, types and frequency of CAM used, patients’ sources of information, causes of their usage, attitudes, and beliefs.

**Results:**

Four hundred currently or previously pregnant women participated. Three hundred fifty-five (91.5%) used at least one method of CAM during pregnancy. Most women used at least one method of biologically-based therapies during pregnancy (87.7%). One hundred and one pregnant women (26.7%) utilised at least one type of herb during pregnancy. The most-reported herb used by 12.3% of participants was anise. Of the mind-body therapies, prayer was the most commonly used method during pregnancy (8.3%). Two hundred and sixty-one (65.3%) participants used CAM because they believed CAM was not harmful to them or their babies during pregnancy. Participants gained most of their information about CAM from their families (43.8%) and friends (24.3%). Approximately two-thirds of participants (64.0%) thought that obstetricians should be able to advise on commonly used CAM.

**Conclusions:**

During pregnancy, CAM products are commonly used, and it is important to determine what types are being used by women in Palestine. These findings supplement the body of knowledge on the use of CAM by pregnant women. Pregnancy care providers need to be aware to the familiar types of CAM that women use.

**Supplementary Information:**

The online version contains supplementary material available at 10.1186/s12906-021-03280-8.

## Background

In pregnant women, the prevalence of complementary and alternative medicines (CAM) is increasing and modalities are widely used in both developing and developed countries [1–3]. The National Institutes of Health categorised CAM into three main groups. The first group, consisting of “Natural Products” that include herbs, minerals, vitamins, and probiotics [4], was considered the most popular CAM group used by American adults in 2012 [4]. The second group was “Mind and Body Practices”, including massage therapy, yoga, chiropractic care, spinal manipulation, osteopathic manipulation, and meditation [5]. The third group includes other complementary health approaches, such as traditional Chinese medicine, traditional healers, homeopathy, Ayurvedic medicine, and naturopathy [4].

Worldwide, CAM is commonly used during pregnancy [1–3]. One study mentioned that almost 50% of pregnant women from different countries outside the USA used at least one CAM type [6]. Surveys from the Middle East found that approximately 75% of pregnant women in Jordan used CAM [7] and 22.3% in Iran used CAM during pregnancy [8]. There has been only one study in Palestine, exploring the use of herbal products during pregnancy [9]. A cross-sectional study conducted by Al-Ramahi et al. [9] found out that 120 women out of 300 (40.0%) used herbal products during pregnancy; most women (82.5%) chose herbs because of their belief that herbs were both more effective and safer than medications.

There has been a small number of studies among the general Palestinian population regarding the use of CAM. For example, several studies focused on CAM in patients with hypertension [10], diabetes mellitus [11], coronary heart disease [12], on haemodialysis [13], cancer [14], or the general public [15]. There have also been several studies on herbal therapies [9, 16–19]. According to these studies, there is a Palestinian interest in the use of CAM, ranging between 45.9–85.9% of the population affected by different types of disease. Some of the types of CAM used in Palestine are common elsewhere [20–22], while other types have been found to be unique to the region [15]. Extensive research on the use of CAM in Palestine found that certain types of CAM, such as honey, prayers, Holy Book reading, al-Hijama, abstinence, and Islamic exorcism, are considered important aspects of culture religion [13, 23–25]**.**

There are many widely used types of CAM available in Palestine, which are used regardless of efficiency or risks. However, there is a lack of knowledge on the use of all types of CAM during pregnancy in Palestine. Only one study explicitly focusing on herbal use during pregnancy has been published from Palestine in 2013 [9]. In addition, many people in Palestine use CAM from traditional or cultural points of view, so we tried to study this phenomenon from a scientific perspective among pregnant women by including all types of CAM in the current study. This study’s aims were: 1) to determine the prevalence of CAM among pregnant women in Palestine, 2) to evaluate the most frequently used CAM therapies during pregnancy, 3) to acknowledge the main sources of data that encourage the use of CAM during pregnancy, and 4) to understand the mindset of pregnant women in Palestine towards the use of CAM. The findings of this study may be helpful in supporting the development of maternal and infant health in the Palestinian community health policy. In addition, the findings of this study could be a basis for safety outcomes, so that pregnant women can be properly advised on CAM, which will eventually direct the use of more safe and effective methods for mother and fetus. Health care providers and policymakers are progressively worried about the use of CAM by pregnant women and the absence of reliable research on the safety and effectiveness of CAM methods [6, 26]. Recognising the patterns of CAM therapy used during pregnancy will allow health care providers to provide more education to pregnant women and help policymakers to build an effective foundation for future policies.

## Methods

### Study design

This study is a cross-sectional, non-interventional, descriptive study of women in Obstetrics and Gynecology (OBGYN) Clinics at Rafedia governmental hospital and in three primary health care clinics in Nablus city, Palestine. This study was conducted during the period of April 2018 to March 2019.

### Setting

This research was carried out in primary health care clinics in Nablus city and in the prenatal clinic/Rafedia Clinic, which is a state clinic in northern Palestine. The OBGYN clinic in Rafedia Hospital treats pregnant women and operates 3 days per week [27].

### Sample size and sampling method

Using the number of currently or previously pregnant women who attended the OBGYN clinic in Nablus district, which number approximately 10,000 pregnant women per year [9, 27], we used the Raosoft online sample size calculator (http://www.raosoft.com/samplesize.html) to measure the sample size, with a pre-determined error margin of 5% and confidence level of 95%. Three hundred seventy pregnant women formed the initial calculated sample size, but the sample size was increased to 400 to account for any missed data or non-response rate to ensure reliability. A convenient sampling technique was used in this study.

### Inclusion and exclusion criteria

Any pregnant woman or woman who has been pregnant for 1 year prior to the survey who visited our health care centers during the period of the study were eligible for inclusion in this study. The exclusion criteria were women who were admitted in an emergency situation, i.e., severe vomiting or elevated blood pressure, or who expressed a lack of willingness to participate in the study.

### Data collection form and instruments

The data were collected using a questionnaire in the native Arabic language, based on similar previous studies [28–32]. The questionnaire was prepared and reviewed by a panel of specialists in the fields of complementary medicine, clinical pharmacy and biostatistics for content validity. Initially, a pilot study was conducted using a primary survey. During this pilot study period, questionnaires were given to only 20 respondents who did not participate in the full study, in the aim of measuring the respondents’ comprehension of the questions. Primary results showed a high prevalence of CAM use and the questionnaire was improved based on the participants’ feedback. An additional file was provided to show a detailed description of the study data collection form in English (Additional file 1).

The questionnaire was structured into five sections:

- The first section included questions regarding the respondents’ socioeconomic and demographic characteristics and reported specifics of age, residency, level of education, body mass index (BMI), monthly family income, and occupation.
- The second section inquired about the participants’ clinical status, medications, habits such as exercise, diet, smoking and alcohol intake, and their sources of information on CAM.
- The third section of the questionnaire focused on participants’ regularity of consumption of CAM and participants were asked to report the kind of CAM used during their pregnancy period. According to previous studies in the same culture, CAM therapies have been classified as follows [13, 23–25], 1) alternative medical systems, such as acupuncture, cupping, Ayurveda and detoxification, (2) biologically-based therapies, such as folic acid, iron, calcium, magnesium, vitamins and other herbs, (3) manipulative and body-based methods, such as massage and manipulation of the spine, (4) mind-body medicine, such as prayer, Quran, religious songs, yoga, meditation, walking or music therapy (exorcism in Islam (ruqya) has been added to mind-body medicine to imitate previous studies), and (5) herbal therapy types. Participants were asked to provide the native name of the herb used. All pregnancy-related herbs and other CAM types to improve or cure health conditions were included in this study.
- In the fourth section, participants were asked to explain the reason(s) for the query [33] that made them use CAM.
- The fifth and last section was designed to assess women’s attitudes and beliefs toward the use of CAM (as adopted from the previously published study) [29], to reveal as many common beliefs of CAM in the Palestinian culture as possible.

### Data collection procedure

Women who were eligible and agreed to participate in the study were asked to fill the surveys by themselves. However, some women who were unable to read or write were interviewed by the researchers to complete their surveys, by oral questioning and writing down their answers. To avoid any biases, the researchers only read the questions for those women and avoided lead questions. Explanations were given to the women when needed to ensure participants understood the questions.

### Ethical approval

Prior to initiation, every aspect of this study protocol was authorised by the Institutional Review Boards (IRB) of An-Najah National University (Archived number: AN5April2018) and the local health authorities.

### Statistical analysis

The Statistical Package for Social Sciences program version 21 (SPSS; New York, USA) was used to enter and analyse data. Four hundred fifty women who had ever experienced pregnancy were invited to participate in this study, and 400 women agreed to complete questionnaires. Descriptive statistics (mean ± standard deviation (SD) or as frequency and percentage) were used to describe categorical variables related to the use of CAM, reasons for CAM usage during pregnancy, types of CAM, types of herbals used and attitudes regarding CAM. In order to see the association between the use of CAM and the characteristics of the participants in the survey, the chi-square test was conducted with a statistically significant *P* value < 0.05.

## Results

### Socio-demographic and obstetric data

During the period of the study, 400 women completed the questionnaire at a response rate of 88.8%. Table 1 displays the distribution of socio-demographic and obstetric features. Of the participants, 168 (42%) were between 25 and 34 years old. One hundred fifty-six (39%) of the subjects were categorised as overweight according to BMI classification (BMI of 25.0–29.9). Two hundred and three (50.8%) were university graduates. Three hundred and twenty-six (81.5%) women were housewives. Approximately 14% of women reported having chronic diseases. One hundred and one women (25.3%) preferred using CAM during pregnancy, and 146 (36.5%) preferred both CAM and medical treatments during pregnancy.

**Table 1:** Socio-demographic and obstetric characteristics of participants (*n* = 400) and their association with CAM use during pregnancy

Variable	n (%)	CAM users n (%) ***N*** = 366	Non-CAM users’ n (%) ***N*** = 34	***P*** value
**Age in years**				
Less than 25	105 (26.3)	101 (27.6)	4 (11.8)	0.013
25–34	168 (42.0)	156 (42.6)	12 (35.3)	
More than 34	127 (31.8)	109 (29.8)	18 (52.9)	
**BMI**				
Underweight	7 (1.8)	6 (1.6)	1 (2.9)	0.919
Normal	154 (38.5)	140 (38.4)	14 (41.2)	
Overweight	156 (39.0)	144 (39.3)	12 (35.3)	
Obese	83 (20.8)	76 (20.8)	7 (20.6)	
**Education**				
Elementary	16 (4.0)	13 (3.6)	3 (8.8)	0.252
Middle school	60 (15.0)	55 (15.0)	5 (14.7)	
High school	121 (30.3)	108 (29.5)	13 (38.2)	
University	203 (50.8)	190 (51.9)	13 (38.2)	
**Residency**				
City	148 (37.0)	134 (36.6)	14 (41.2)	0.614
Village	225 (56.3)	206 (56.3)	19 (55.9)	
Palestinian refugee camps	27 (6.8)	26 (7.1)	1 (2.9)	
**Monthly income**				
Less than 2000 NIS	144 (36.0)	129 (35.3)	15 (44.1)	0.194
2000–5000 NIS	222 (55.5)	208 (57.0)	14 (41.2)	
5000–10,000 NIS	24 (6.0)	21 (5.8)	3 (8.8)	
More than 10,000 NIS	9 (2.3)	7 (1.9)	2 (5.9)	
**Occupation**				
Housewife	326 (81.5)	301 (82.2)	25 (73.5)	0.421
Governmental employee	45 (11.3)	40 (10.9)	5 (14.7)	
Private sector employee	29 (7.2)	25 (6.8)	4 (11.8)	
**Birthplace**				
Palestine	366 (91.5)	335 (91.5)	31 (91.2)	0.944
Abroad	34 (8.5)	31 (8.5)	3 (8.8)	
**Number of children**				
Pregnant (0)	44 (11.0)	39 (10.7)	5 (14.7)	0.513
1–3	213 (53.3)	199 (54.4)	14 (41.2)	
4–6	118 (29.5)	106 (29.0)	12 (35.3)	
> 6	25 (6.2)	22 (6.0)	3 (8.8)	
**Presence of chronic diseases**				
Yes	56 (14.0)	45 (12.3)	11 (32.4)	0.001
No	344 (86.0)	321 (87.7)	23 (67.6)	
**Treatment preference**				
Medical	153 (38.3)	139 (38.0)	14 (41.2)	0.143
CAM	101 (25.3)	97 (26.5)	4 (11.8)	
Both	146 (36.5)	130 (35.5)	16 (47.1)	

Abbreviations: *CAM* complementary and alternative medicine, *BMI* body mass index, *NIS* New Israeli Shekel (1 New Israeli Shekel = 0.29 US Dollar)

### Sources of information about CAM

Table 2 shows that pregnant women gained most of their information about CAM during pregnancy and during their lifetime from their families (43.8%) and friends (24.3%).

**Table 2:** Sources of information about CAM use for the entire study participants

Source	n (%)^**a**^
Physician	54 (13.5)
Midwife/nurse	18 (4.5)
Pharmacist	9 (2.3)
Television	46 (11.5)
Radio	22 (5.5)
Family	175 (43.8)
Friends	97 (24.3)
Neighbours	75 (18.8)
Social media	69 (17.3)

^a^Total percentages exceed 100% due to the selection of more than one source

### Prevalence of CAM use during pregnancy and during their lifetime

A total of 400 women who participated (100%) reported using at least one method of CAM during their lifetime, with 355 (91.5%) of them reported use of at least one method during pregnancy, as shown in Tables 3 and 4. A comparison of the characteristics of CAM users and non-CAM users during pregnancy revealed that no significant difference (*p*-value > 0.05) occurred in all sociodemographic and clinical characteristics, except in terms of age and prevalence of chronic diseases, which showed a significant difference (*p*-value < 0.05); (Table 1). According to the women’s age, women who were older than 34 years old used CAM significantly less than those who were less than 34 years old (29.8% versus 52.9%, *p*-value = 0.013). In addition, women with chronic diseases used CAM significantly less than those who did not have chronic diseases (12.3% versus 87.7%, *p*-value = 0.001); (Table 1).

**Table 3:** CAM categories, their frequencies and percentages of use during pregnancy and during the lifetime

CAM Categories	During pregnancy n (%)	Overall n (%)
Mind-body medicine	72 (18.0)	371 (92.8)
Alternative medical systems	13 (3.3)	62 (15.5)
Manipulative and body-based methods	34 (8.5)	161 (40.3)
Biologically-based therapies	351 (87.8)	382 (95.5)
Herbal	107 (26.8)	392 (98.0)

**Table 4:** Types of CAM of each category used, their frequencies and percentages during pregnancy and during the lifetime

Types of CAM	During pregnancy n (%)	In general n (%)	Never used it n (%)
**Mind-body medicine**			
Prayer	33 (8.3)	304 (76.0)	63 (15.8)
Quran	29 (7.3)	324 (81.9)	47 (11.8)
Religious songs	9 (2.3)	115 (28.8)	276 (69.0)
Exorcism	4 (1.0)	13 (3.3)	383 (95.8)
Yoga	9 (2.3)	15 (3.8)	376 (94.0)
Meditation	22 (5.5)	64 (16.0)	314 (78.5)
Dancing	9 (2.3)	66 (16.5)	325 (81.3)
Music	8 (2.0)	69 (17.3)	323 (80.8)
Hypnotherapy	2 (0.5)	5 (1.3)	393 (98.3)
Aroma therapy	3 (0.8)	15 (3.8)	382 (95.5)
**Alternative medical systems**			
Acupuncture	12 (3.0)	7 (1.8)	381 (95.3)
Cupping	2 (0.5)	34 (8.5)	364 (91.0)
Ayurveda	1 (0.3)	1 (0.3)	398 (99.5)
Detox	1 (0.3)	17 (4.3)	382 (95.5)
**Manipulative and body-based methods**			
Spine manipulation	4 (1.0)	9 (2.3)	387 (96.8)
Massage	32 (8.0)	127 (31.8)	241 (60.3)
**Biologically-based therapies**			
Vitamin D	153 (38.3)	67 (16.8)	180 (45.0)
Vitamin C	163 (40.8)	54 (13.5)	183 (45.8)
Folic acid	305 (76.3)	23 (5.8)	72 (18.0)
B complex	110 (27.5)	64 (16.0)	226 (56.5)
Calcium	212. (53.0)	47 (11.8)	141 (35.3)
Magnesium	72 (18.0)	32 (8.0)	296 (74.0)
Iron	290 (72.5)	46 (11.5)	64 (16.0)
Prebiotics	9 (2.3)	11 (2.8)	380 (95.0)
Probiotics	8 (2.0)	10 (2.5)	382 (95.5)
Glutamine	3 (0.8)	5 (1.3)	392 (98.0)
Glycine	4 (1.0)	4 (1.0)	392 (98.0)
Carnitine	2 (0.5)	2 (0.5)	396 (99.0)
Arginine	3 (0.8)	3 (0.8)	394 (98.5)
Fish oil	39 (9.8)	95 (23.8)	266 (66.5)
Omega 3	43 (10.8)	45 (11.3)	312 (78.0)
Soy	4 (1.0)	17 (4.3)	379 (94.8)

### Types of CAM used during pregnancy and during their lifetime

Biologically-based therapies were the most commonly used category during pregnancy by 351 (87.8%) women, as shown in Table 3. It is also apparent from Table 3 that biologically-based medicines, excluding herbs, were reported to be used by 382 (95.5%) of the participants during their lifetime. Table 4 displays that prayer was the most common mind-body medicine method used during pregnancy (8.3%). An alternative medical system was used only by 62 participants (15.5%). The massage was the most prominent manipulation and body-based method by 32 (8%) participants during pregnancy. It is also apparent from Table 4 that folic acid (76.3%) and iron (72.5%) were mostly used during pregnancy.

### Types of herbs used during pregnancy and during their lifetime

Table 5 provides frequencies of herbal use, which 392 participants (98%) reported using during their lifetime. It is also evident from Table 5 that 107 (26.8%) of the participants during pregnancy were reported to use herbs. During pregnancy, the top five herbs used by pregnant women were anise (12.3%), peppermint (11.5%), sage (10.8%), chamomile (9.5%), and cinnamon (9%).

**Table 5:** Types of herbals used, their frequencies and percentages during pregnancy and during the lifetime

Types of Herbals	During pregnancy n (%)	In general n (%)	Never used it n (%)
Sage (*Lamiaceae Salvia officinalis L.)*	43 (10.8)	292 (73.0)	65 (16.3)
Thyme (*Lamiaceae Thymus vulgaris L.)*	33 (8.3)	264 (66)	103 (25.8)
Peppermint (*Lamiaceae Mentha piperita L.)*	46 (11.5)	290 (72.5)	64 (16.0)
Fenugreek (*Leguminosae Trigonella foenum-graecum L)*	21 (5.3)	166 (41.5)	213 (53.3)
Chamomile (*Asteraceae Matricaria chamomilla L)*	38 (9.5)	269 (67.3)	93.0 (23.3)
Anise (*Apiaceae Pimpinella anisum L.)*	49 (12.3)	289 (72.3)	62 (15.5)
Ginger (*Zingiberaceae Zingiber officinale Roscoe)*	21 (5.3)	182 (45.5)	197 (49.3)
Cumin (*Apiaceae Cuminum cyminum L)*	28 (7.0)	187 (46.8)	185 (46.3)
Cinnamon (*Lauraceae Cinnamomum verum J. Presl)*	36 (9.0)	215 (53.8)	149 (37.3)
Turmeric (*Zingiberaceae Curcuma longa)*	8 (2.0)	132 (33.0)	260 (65.0)
Garlic (*Amaryllidaceae Allium sativum L)*	18 (4.5)	191 (47.8)	191 (47.8)
Parsley (*Apiaceae Petroselinum crispum (Mill.)*	27 (6.8)	215 (53.8)	158 (39.5)
Green tea (*Theaceae Camellia sinensis (L.)*	11 (2.8)	150 (37.5)	239 (59.8)
Saffron (*Iridaceae Crocus sativus)*	6 (1.5)	71 (17.8)	323 (80.8)
Rosemary (*Rosmarinus officinalis)*	7 (1.8)	145 (36.3)	248 (62.0)
Ginseng (*Araliaceae Panax ginseng*)	2 (0.5)	30 (7.5)	368 (92.0)
Olive oil (*Oleaceae Olea europaea)*	27 (6.8)	286 (71.5)	87 (21.5)
Castor oil (*Euphorbiaceae Ricinus communis L)*	18 (4.5)	166 (41.5)	216 (54.0)
Grapeseed oil (*Vitis vinifera seed oil)*	1 (0.3)	23 (5.8)	376 (94.0)
Almond oil (*Prunus amygdalus L.)*	2 (0.5)	56 (14.0)	342 (85.5)
*Aloe vera* (*Aloe perfoliata L.)*	3 (0.8)	61 (15.3)	336 (84.0)
Cranberry (*Vaccinium macrocarpon*)	5 (1.3)	47 (11.8)	348 (87.0)

### Reasons for CAM usage during pregnancy

As shown in Table 6, 42% of participants agreed that CAM is more effective than medical therapy, although 39.5% did not agree with this statement. Two hundred and sixty-one (65.3%) participants believed it was not harmful to them or their babies during pregnancy. Only 84 (21.0%) of the participants reported that they used CAM because medical therapies failed to succeed. Two hundred and fifty-two (63.0%) subjects used CAM based on others’ experiences. Two hundred and thirty-one (57.8%) used CAM because it was more accessible, compared to medical therapy. Also, 262 (65.5%) of the participants used CAM because of its common use and recommendation in their culture. Although not specific to pregnancy, most participants used a CAM modality to relieve influenza symptoms (84.0%), and 273 (68.3%) of the respondents have used it to support medical therapy. Two hundred and sixty-nine (67.3%) used CAM because they thought it could be beneficial during pregnancy.

**Table 6:** Reasons for CAM usage during pregnancy

Item^**a**^	Agree n (%)	Disagree n (%)	No opinion n (%)
“It is more effective than medical therapy.”	168 (42.0)	158 (39.5)	74 (18.5)
“It is because it is not harmful for you and your baby during pregnancy.”	261 (65.3)	86 (21.5)	53 (13.3)
“It is because Medical therapies failed to succeed.”	84 (21.0)	281 (70.3)	35 (8.8)
“It is because Others have tried it and were satisfied.”	252 (63.0)	124 (31.0)	24 (6.0)
“It is because It is more accessible compared to medical therapy.”	231 (57.8)	139 (34.8)	30 (7.5)
“It is because It is used and recommended in our culture.”	262 (65.5)	105 (26.3)	33 (8.3)
“You have used it in order to relieve symptoms of illnesses such as common cold and influenza.”	336 (84.0)	48 (12.0)	16 (4.0)
“You have used it in order to support medical therapy”.	273 (68.3)	78 (19.5)	49 (12.3)
“It is because you think It can be beneficial during pregnancy.”	269 (67.3)	83 (20.8)	48 (12.0)

^a^Questions were adapted from Koç Z et al., [33]

### The attitude of women toward CAM usage during pregnancy

Table 7 provides results that reflect pregnant women’s attitudes and beliefs about CAM usage during pregnancy, according to their experiences. Two hundred and fifty (62.5%) women agreed that CAM gave them more health and control over their bodies. One hundred and eighty-one (45.5%) of the participants thought that CAM was a better preventative measure than medical therapy. Two hundred and thirty-five (58.8%) participants believed that CAM promoted a holistic approach to health. Two hundred and forty-six (61.5%) believed that proof of effectiveness was important to their choice of CAM. One hundred and ninety-nine (49.8%) reported that their experiences with CAM’s effectiveness were more vital than clinical evidence. Of the participants, 256 (64.0%) thought that obstetricians should be able to advise their patients on commonly used CAM.

**Table 7:** Pregnant women’s attitudes towards CAM usage in pregnancy

Item^**a**^	Agree n (%)	Disagree n (%)	No opinion n (%)
“CAM gives me more control over my health/body.”	250 (62.5)	64 (16.0)	86 (21.5)
“CAM is a better preventative measure than CM.”	181 (45.5)	117 (29.3)	102 (25.5)
“CAM promotes a holistic approach to health.”	235 (58.8)	92 (23.0)	73 (18.3)
“Evidence of effectiveness is important to my choice of CAM.”	246 (61.5)	78 (19.5)	76 (19.0)
“My personal experience of the effectiveness of CAM is more important than clinical evidence.”	199 (49.8)	113 (28.3)	88 (22.0)
“Obstetricians should be able to advise their patients about commonly used CAM.”	256 (64.0)	51 (12.8)	93 (23.3)

Abbreviations: *CM* conventional medicine, *CAM*,complementary and alternative medicine

^a^Questions were adapted from Frawley et al., [29]

## Discussion

The current study found that at least one method of CAM was used by 91.5% of Palestinian women during their entire pregnancy period, which is significantly higher than other studies conducted in the USA (67%) [34], the UK (57.1%) [35], Turkey (41.4%) [33], Jordan (75%) [36], Australia (52%) [28] and Germany (50%) [37].

To our knowledge, this is the first study in Palestine that investigates all types of CAM use during pregnancy and women’s attitudes on CAM use in pregnancy. Our study was planned to cover all forms of CAM methods used for the sole purpose of treating health problems during pregnancy; however, only one previous study in Palestine [9] was conducted, which investigated the use of only herbal products during pregnancy. We found that the most common cause of CAM usage during pregnancy was to relieve symptoms of influenza, which, though not specific for pregnancy, participants believed using CAM during pregnancy is safer than taking medication to treat conditions such as the simple flu. This particular finding is consistent with the results of a previous study performed in Palestine [9]. Two-thirds (65.3%) of participants believed that CAM is not harmful to themselves or their babies during pregnancy, which is comparable to results from a study from Turkey (69.2%) [33]. It was, however, higher than results of studies conducted in Iran (39.8%), the UK (19%), Nigeria (29.0%) and Iraq (43%) [8, 36, 38, 39].

As for mind-body medicine, prayer was the most common method used by Palestinian women during their pregnancy periods. This reflects the effect of religion and culture on people’s lifestyles during pregnancy and their lifetimes in Palestine’s West Bank [15].

Another important finding shows that most pregnant women used vitamins and minerals, mostly folic acid (76.3%) and iron (72.5%), which is similar to results from a study conducted in Australia [40]. One possible explanation is that most pregnant women follow the instructions and recommendations of their health care provider. In the same study, it is apparent that women in Palestine use calcium twice as much as women in Australia during pregnancy. In Australia, it has been reported that the most common cause of calcium use was a doctor-recommended dietary supplement [40].

Results of herbal usage in our study are lower than in studies done in the UK (59%) [38], Egypt (27.3%) [41], Nigeria (67.5%) [39], Iraq (53.7%) [36] and Norway (39.7%) [42], but higher than Iran (22.3%) [8] and Taiwan (20.2%) [43]. The results of this study show that herbs are used by 26.8% of women during pregnancy, which is less than the study conducted in Palestine in 2013, which showed that 40% of women used herbs during pregnancy [9]. This discrepancy in results could be attributed to the fact that we interviewed pregnant and previously pregnant women, inferring that the subjects might not recall exactly what they used in previous pregnancies, in comparison to the 2013 study, in which postpartum women were interviewed within 3 days of their delivery.

The most common herbs used were found to be anise, peppermint, sage, chamomile, and cinnamon which is consistent with the study conducted in Palestine in 2013, in which anise and chamomile were the most commonly reported herbs used by pregnant women in that country [9] as well as Egypt [41], and peppermint in Turkey [33]. While ginger was found to be the most reported herb used in the UK and Norway [38, 42], red raspberry leaf is one of the most widely used herbal teas during pregnancy in other countries, mainly due to its presumed effect on labour stimulation [44, 45]. This variation in herbs use in different countries may be due to the use of these herbs in standard dietary ingredients as a spice, a flavouring agent or to reduce food spoilage in different cultures [46–49].

Most women in Palestine believe that anise helps to facilitate labour, which is a belief that refers to traditions. There is currently no evidence, however, suggesting that anise helps to facilitate labour. As we found in our research, most CAM-related knowledge was based on tradition instead of evidence. The findings of systematic reviews and meta-analyses [50] suggest that herbal medicines for induction of labour are effective, however, due to the lack of good quality data, there exists inconclusive safety evidence [50, 51].

The current study results found that some herbs used by pregnant women are considered undesirable to be used during pregnancy. For example, sage in large doses is better to be avoided for concern of potential miscarriage effect. Concentrated anise has a concern that it might trigger early labour. And thyme in large doses is better to be avoided, especially in early pregnancy, because of concern of potential miscarriage [52].

Regarding the attitude towards CAM usage in pregnancy, this study found that almost half (49.8%) of women who participated in the study believed that their personal experiences regarding the effectiveness of CAM were enough. For them, the experiences were more important and significant than clinical evidence. Although a majority (61.5%) of participants reported a need for evidence of effectiveness to use any method of CAM, this evidence is probably the result of traditional use and not scientific evidence. This could be explained by the most common sources of information on using CAM during pregnancy, which were their families (43.8%) and friends (24.3%). This attitude of pregnant women in Palestine reflects the fact that 42% of study subjects believed that CAM was more effective than medical therapy. This indicates a possible lack of trust in medical consultations and services in Palestine, as similarly found by Frawley et al. [29], raising concerns due to lack of data regarding women’s’ beliefs of CAM usage safety during pregnancy.

This study’s findings provide valuable information for policy makers in Palestine regarding pregnant women’s use of and attitudes towards CAM, which can be used to raise awareness of health care providers who should be able to increase awareness and knowledge of risks and benefits of CAM to mothers. The authors also believe that health care providers should be the only source of information regarding CAM, prompting a recommendation to introduce CAM as part of the medical degree syllabus.

### Strengths and limitations

Our study has multiple points of strength, including powered sample size and multi-centered data collection. Nonetheless, these results must be interpreted with care and a number of limitations should be considered since the effect estimates in the model are based on a cross-sectional study. The survey data is self-reported and is therefore vulnerable to bias recall. Since the survey was conducted for pregnant women or women who have been pregnant for 1 year prior to survey performance, recall bias would be minimal. It is unfortunate that our study could not identify the information sources for undesirable and desirable CAM use.

## Conclusions

Usage of CAM among pregnant women in Palestine is significantly high, as this study shows. The most commonly used methods are biologically-based therapies and herbs, as found in other countries. Most participants considered CAM safe to use in pregnancy and almost half find it more effective than medical therapy. This new body of knowledge can inform health care providers regarding the need to discuss the risks and benefits of using CAM modalities during pregnancy to ensure women make an informed choice before using such treatments. Furthermore, the current study should guide and inspire investigators to conduct CAM-related medical research in order to provide stronger evidence of possible CAM-related benefits and risks. Additional CAM-based clinical research, i.e. prospective cohort study is required to obtain clearer data regarding the possible benefits and risks of the use of CAM. In addition, further research is also required to identify CAM modality that is considered undesirable to be used during pregnancy.

## Supplementary Information

**Additional file 1:.** Study questionnaires. This is the final version of the questionnaire in English used to evaluate the use of CAM during pregnancy.

## Data Availability

The datasets used for the current study are available from the corresponding author upon request.

## Abbreviations

IRB: Institutional Review Board
CAM: Complementary and alternative medicine
SPSS: Statistical Package for the Social Sciences
OBGYN: Obstetrics and gynecology
BMI: Body mass index
SD: Standard deviation
