# Supplementary material for: The use of complementary and alternative medicine during pregnancy: a cross-sectional study from Palestine
Source: BMC Complement Med Ther. 2021 Apr 1;21:108. doi: 10.1186/s12906-021-03280-8 (PMC8017862; doi:10.1186/s12906-021-03280-8)
Supplement: Supplementary file 1 — Additional file 1:. Study questionnaires. This is the final version of the questionnaire in English used to evaluate the use of CAM during pregnancy. [file 12906_2021_3280_MOESM1_ESM.doc]

**Study questionnaires:** This is the final version of the English version that was used to evaluate the use of complementary and alternative medicine during pregnancy

| **Part one:** | | | | | | | | |
| --- | --- | --- | --- | --- | --- | --- | --- | --- |
| **Age:** | |  | | | | | | |
| **Height:** | | | | | | | | |
| **Weight:** | | | | | | | | |
| **Residency:** | 1.City | | 2.Village | | 3.Palestinian refugee camp | | | |
| **Education level:** | 1.Elementary | | 2.Middle school | | 3.High school | | | 4.University |
| **Occupation:** | 1.Housewife | | 2.Governmental employee | | | 3.Private sector  employee | | |
| **Monthly income:** | 1. Less than 2000 NIS | | 2. 2000-5000 NIS | 3. 5000-10000 NIS | | | 4. More than 10000 NIS | |
| **Health insurance** | 1.No insurance | | 2.Governmental insurance | 3.Private insurance | | | | |
| **Birth place:** | 1.Palestine | | 2.Abroad | | | | | |
| **Number of Children:** | | | | | | | | |

| **Part two:** | | | |
| --- | --- | --- | --- |
| **Do you have any chronic diseases?** | - No | | - Yes - Cardiovascular disease - Thyroid disease - Hypertension - Diabetes Mellitus - Renal failure - Epilepsy - Asthma - Thalassemia - Other(mention): |
| **Do you use any drugs for your chronic disease?** | - No | | - Yes(mention): |
| **Do you exercise regularly?** | - No | | - Yes (mention): |
| **Do you smoke?** | - No | | - Yes |
| **Do you drink alcohol?** | - No | | - Yes |
| **What type of treatment do you prefer to use during pregnancy?** | - Medical treatment | - Complementary and alternative Medicine (CAM) | - Both |
| **Did someone advise you to use CAM?** | - No | | - Yes - Doctor - Midwife/nurse - Pharmacist - Family - Friends - Neighbors - Tv - Radio - Social media |
| **Do you follow any specific diet?** | - No | | - Yes - Vegan/vegetarian - Weight reduction - Low calorie - Low carbohydrates - Low fat - For medical reason |

| **Part three:** | | | |
| --- | --- | --- | --- |
| **Type of CAM** | **Used in pregnancy**  **(1)** | **Used in general**  **(2)** | **Never used**  **(3)** |
| - **Mind-body medicine** | | | |
| Prayer | 1 | 2 | 3 |
| Quran | 1 | 2 | 3 |
| Religious songs | 1 | 2 | 3 |
| Exorcism | 1 | 2 | 3 |
| Yoga | 1 | 2 | 3 |
| Meditation | 1 | 2 | 3 |
| Dancing | 1 | 2 | 3 |
| Music | 1 | 2 | 3 |
| Hypnotherapy | 1 | 2 | 3 |
| Aromatherapy | 1 | 2 | 3 |
| - **Alternative medical system** | | | |
| Acupuncture | 1 | 2 | 3 |
| Cupping | 1 | 2 | 3 |
| Ayurveda | 1 | 2 | 3 |
| Detox | 1 | 2 | 3 |
| - **Manipulative and Body-Based Methods** | | | |
| Spine manipulation | 1 | 2 | 3 |
| Massage | 1 | 2 | 3 |
| - **Biologically Based Therapies** | | | |
| Vitamin D | 1 | 2 | 3 |
| Vitamin C | 1 | 2 | 3 |
| Folic acid | 1 | 2 | 3 |
| B complex | 1 | 2 | 3 |
| Calcium | 1 | 2 | 3 |
| Magnesium | 1 | 2 | 3 |
| Iron | 1 | 2 | 3 |
| Prebiotic | 1 | 2 | 3 |
| Probiotic | 1 | 2 | 3 |
| Glutamine | 1 | 2 | 3 |
| Glycine | 1 | 2 | 3 |
| Carnitine | 1 | 2 | 3 |
| Arginine | 1 | 2 | 3 |
| Fish oil | 1 | 2 | 3 |
| Omega 3 | 1 | 2 | 3 |
| Soy bean | 1 | 2 | 3 |
| - **Herbals** | | | |
| Sage | 1 | 2 | 3 |
| Thyme | 1 | 2 | 3 |
| Peppermint | 1 | 2 | 3 |
| Fenugreek | 1 | 2 | 3 |
| Chamomile | 1 | 2 | 3 |
| Anise | 1 | 2 | 3 |
| Ginger | 1 | 2 | 3 |
| Cumin | 1 | 2 | 3 |
| Cinnamon | 1 | 2 | 3 |
| Turmeric | 1 | 2 | 3 |
| Garlic | 1 | 2 | 3 |
| Parsley | 1 | 2 | 3 |
| Green tea | 1 | 2 | 3 |
| Saffron | 1 | 2 | 3 |
| Rosemary | 1 | 2 | 3 |
| Ginseng | 1 | 2 | 3 |
| Olive oil | 1 | 2 | 3 |
| Castor oil | 1 | 2 | 3 |
| Grapeseed oil | 1 | 2 | 3 |
| Almond oil | 1 | 2 | 3 |
| Alovera | 1 | 2 | 3 |
| Cranberry | 1 | 2 | 3 |

| **Part four:** | | | |
| --- | --- | --- | --- |
| **Reasons of CAM usage in pregnancy #** | **Agree**  **(1)** | **Disagree**  **(2)** | **No opinion**  **(3)** |
| “It is more effective than medical therapy.” | 1 | 2 | 3 |
| “It is because it is not harmful for you and your baby during pregnancy.” | 1 | 2 | 3 |
| “It is because Medical therapies failed to succeed.” | 1 | 2 | 3 |
| “It is because Others have tried it and were satisfied.” | 1 | 2 | 3 |
| “It is because It is more accessible compared to medical therapy.” | 1 | 2 | 3 |
| “It is because It is used and recommended in our culture.” | 1 | 2 | 3 |
| “You have used it in order to relieve symptoms of illnesses such as common cold and influenza.” | 1 | 2 | 3 |
| “You have used it in order to support medical therapy”. | 1 | 2 | 3 |
| “It is because you think It can be beneficial during pregnancy.” | 1 | 2 | 3 |

# These questions were adapted from Koç Z, Sağlam Z, Topatan S: **Determination of the usage of complementary and alternative medicine among pregnant women in the Northern Region of Turkey**. *Collegian* 2017, **24**(6):533-539.

| **Part five:** | | | |
| --- | --- | --- | --- |
| **Your attitude towards CAM usage in pregnancy#** | **Agree**  **(1)** | **Disagree**  **(2)** | **No opinion**  **(3)** |
| “CAM gives me more control over my health/body.” | 1 | 2 | 3 |
| “CAM is a better preventative measure than CM.” | 1 | 2 | 3 |
| “CAM promotes a holistic approach to health.” | 1 | 2 | 3 |
| “Evidence of effectiveness is important to my choice of CAM.” | 1 | 2 | 3 |
| “My personal experience of the effectiveness of CAM is more important than clinical evidence.” | 1 | 2 | 3 |
| “Obstetrics and gynecologists should be able to advise their patients about commonly used CAM.” | 1 | 2 | 3 |

# These questions were adapted from Frawley J, Sibbritt D, Broom A, Gallois C, Steel A, Adams J: **Women's attitudes towards the use of complementary and alternative medicine products during pregnancy**. *J Obstet Gynaecol* 2016, **36**(4):462-467.
